# Development and Validation of an Insulin Resistance Predicting Model Using a Machine-Learning Approach in a Population-Based Cohort in Korea

**DOI:** 10.3390/diagnostics12010212

**Published:** 2022-01-16

**Authors:** Sunmin Park, Chaeyeon Kim, Xuangao Wu

**Affiliations:** 1 Department of Food and Nutrition, Obesity/Diabetes Research Center, Hoseo University, Asan 31499, Korea; 76txt@naver.com; 2 Department of Bio-Convergence System, Hoseo University, Asan 31499, Korea; niyani0@naver.com

**Keywords:** insulin resistance, HOMA-IR, machine learning, XGboost, liver function, obesity

## Abstract

Background: Insulin resistance is a common etiology of metabolic syndrome, but receiver operating characteristic (ROC) curve analysis shows a weak association in Koreans. Using a machine learning (ML) approach, we aimed to generate the best model for predicting insulin resistance in Korean adults aged > 40 of the Ansan/Ansung cohort using a machine learning (ML) approach. Methods: The demographic, anthropometric, biochemical, genetic, nutrient, and lifestyle variables of 8842 participants were included. The polygenetic risk scores (PRS) generated by a genome-wide association study were added to represent the genetic impact of insulin resistance. They were divided randomly into the training (*n* = 7037) and test (*n* = 1769) sets. Potentially important features were selected in the highest area under the curve (AUC) of the ROC curve from 99 features using seven different ML algorithms. The AUC target was ≥0.85 for the best prediction of insulin resistance with the lowest number of features. Results: The cutoff of insulin resistance defined with HOMA-IR was 2.31 using logistic regression before conducting ML. XGBoost and logistic regression algorithms generated the highest AUC (0.86) of the prediction models using 99 features, while the random forest algorithm generated a model with 0.82 AUC. These models showed high accuracy and k-fold values (>0.85). The prediction model containing 15 features had the highest AUC of the ROC curve in XGBoost and random forest algorithms. PRS was one of 15 features. The final prediction models for insulin resistance were generated with the same nine features in the XGBoost (AUC = 0.86), random forest (AUC = 0.84), and artificial neural network (AUC = 0.86) algorithms. The model included the fasting serum glucose, ALT, total bilirubin, HDL concentrations, waist circumference, body fat, pulse, season to enroll in the study, and gender. Conclusion: The liver function, regular pulse checking, and seasonal variation in addition to metabolic syndrome components should be considered to predict insulin resistance in Koreans aged over 40 years.

## 1. Introduction

Insulin acts by binding to the insulin receptors to activate the insulin-receptor substrates (IRS) via phosphorylation in various tissues [1,2]. IRS phosphorylation induces the signaling cascades through PI3-kinase and protein kinase B activation to improve glucose uptake into the tissues, glycogenesis, lipogenesis, and protein synthesis [3]. When the blood glucose levels are elevated, insulin is released from the pancreatic β-cells to maintain glucose homeostasis. Insulin resistance is defined as the condition with attenuated insulin signaling in various tissues, particularly skeletal muscles, adipose tissues, and the liver, to elevate insulin secretion to make normoglycemia [4]. People with insulin resistance have hyperinsulinemia and develop prediabetic conditions in Caucasians [3,4]. However, Asians do not develop hyperinsulinemia when insulin resistance occurs because of the small pancreatic β-cell mass and low insulin secretion capacity. Prediabetes progresses quickly to type 2 diabetes in Asians, accelerating cardiovascular disease progression [5,6].

Genetic and environmental factors affect insulin resistance, contributing to developing metabolic diseases. Genetic factors are highly associated with insulin secretion capacity along with insulin sensitivity under the insulin-resistant state, especially in Asians [7,8]. Environmental factors mainly included aging, sedentary lifestyle, alcohol drinking, smoking, unhealthy eating habits, including high intake of refined sugar and animal fat and low intake of dietary fiber, vitamin C, vitamin D, and calcium [9,10]. These factors interact with various biochemical parameters involved in energy, glucose, lipid, and immune metabolism to modulate insulin resistance.

Insulin resistance can be measured directly using a hyperinsulinemic-euglycemic clamp, but it is conducted for clinical studies, not in a clinical setting. The homeostasis model assessment of insulin resistance (HOMA-IR) is an indirect insulin resistance index altered according to age and gender in the Korean population [11]. Insulin resistance is a common etiology for metabolic syndrome (MetS), represented by a cluster of abdominal obesity, hyperlipidemia, hyperglycemia, and hypertension, which are interrelated to the attenuation of insulin signaling [12]. However, insulin resistance is weakly linked to MetS after adjusting for gender and age in the receiver operating characteristic (ROC) curve analysis (area under the curve (AUC) of the ROC curve = 0.67). Moreover, the MetS components were also associated with insulin resistance (AUC = 0.614–0.75) in Korean population studies [5]. Thus, it is better to find better risk factors to predict insulin resistance in a clinical setting. The cutoff values for HOMA-IR vary according to age, gender, and metabolic disease status. The cutoff value of HOMA-IR for MetS risk in the Korean National Health and Nutrition Examination Survey (KNHANES) during 2008–2010 is 2.11 for men, 2.0 for premenopausal women, and 2.14 for post-menopause women [11]. On the other hand, the HOMA-IR for the dysglycemia risk in KNHNAES in 2015 is 1.6 in both genders, and for the type 2 diabetes risk, the cutoff value is 2.9 for men and 2.4 for women [5]. The cutoff value of HOMA-IR for normoglycemia and prediabetes in different populations is 1.8–2.5 [13,14]. Therefore, the cutoff for insulin resistance should be assigned to the study population. 

Insulin resistance can be used for the early detection of MetS, type 2 diabetes, nonalcoholic fatty liver disease (NAFLD), nonalcoholic steatohepatitis (NASH), and cardiovascular diseases [15]. Although the risk factors for insulin resistance are well known, there is no suitable model for insulin resistance to explain the etiology and predict it in Asians. The machine learning approach to disease diagnosis and prediction has increased recently. The AUC of the ROC curve is applied for external validation of the optimal fitting of the model, and the target values are 0.8–0.85, suggesting that the model is adequate for predicting insulin resistance [16,17]. The prediction model can identify the insulin resistance risk factors to predict cardiometabolic diseases early. When the insulin resistance prediction model is available, the model can be applied to classify the participants into low- and high-insulin resistance groups in the large cohorts in the Korean Genomic and Epidemiological Research Study (KoGES), which did not include the HOMA-IR values, and the genetic impacts and gene-lifestyle interaction can be studied. Therefore, the objective of the present study was to generate the best predicting model for insulin resistance, defined by HOMA-IR, from genetic, environmental, and biochemical factors using a machine-learning algorithm in an Ansan/Ansung cohort. This study also provided crucial risk factors for insulin resistance in Asians. 

## 2. Materials and Methods

### 2.1. Participants 

KoGES included the Ansan/Ansung, rural, and city hospital-based cohorts. However, serum insulin concentrations were measured only in the Ansan/Ansung cohort to estimate the insulin resistance index. The participants were aged between 40 and 74 years and were residents of Ansan City (a large city area; *n* = 4205) or Ansung City (a small city area; *n* = 4637) from 2001 to 2007. The Institutional Review Board of the Korean National Institute of Health approved the KoGES (KBP-2015-055), and Hoseo University approved the present study (1041231-150811-HR-034-01). Written informed consent was obtained from all subjects. 

### 2.2. Demographic, Anthropometric, and Biochemical Measurements

The participants who lived within the Ansan/Ansung areas for at least six months participated voluntarily in the cohort study. The participants with a severe stage of cancer and metabolic diseases were excluded. The demographic information, including age, education, income, smoking history, alcohol consumption, and physical activity, was collected in a health interview. 

The height, weight, and waist and hip circumference were measured in patients wearing a light gown. The body fat and muscle mass were estimated by bioelectrical impedance analysis (Inbody 3.0, Biospace, Seoul, Korea) [18]. The body mass index (BMI) was calculated from the weight [kg]/square of height [m^2^]. Lean body mass and fat mass were measured by Inbody 4.2 (Cheonan, Korea). Skeletal muscle mass index was calculated by dividing lean body mass by body weight × 100, while body fat percent was determined by dividing total fat mass by body weight × 100. The blood pressure was determined on the right arm at the same height as their heart in the sitting and lying positions three times, and the average values were used. The pulse was also counted before assessing the blood pressure. Current smokers were defined as having smoked more than 100 cigarettes throughout their lifetime and within the last six months, whereas former smokers had not smoked for the last six months. Alcohol intake was assessed by the drinking frequencies and the alcohol amount in each drinking event during the last six months prior to the interview. The regular activity was evaluated as a regular moderate exercise for >30 min at a time at least five times a week or as regular vigorous exercise for >20 min at one time at least three times per week. 

The blood samples from each participant were collected after an overnight fast, and the serum and plasma were separated. The biochemical variables were measured using an automatic analyzer (ZEUS 9.9; Takeda, Tokyo, Japan). The variables were as follows: glucose, total cholesterol, HDL-cholesterol, triglycerides, platelet, alanine aminotransferase (ALT), aspartate aminotransferase (AST), γ-glutamyl transpeptidase (γ-GTP), creatinine, and total bilirubin. Fasting serum insulin levels and high-sensitive C-reactive protein (CRP) were analyzed using ELISA kits (DiaSorin, Stillwater, MN, USA). Serum LDL concentrations were calculated with the Friedewald formula: serum total cholesterol–serum HDL–serum triglyceride/5. The estimated glomerular filtration rate (eGFR) was estimated using the equation of 175 × (serum creatinine concentrations)^−1.154^ × (age)^−0.203^. In females, the eGFR was multiplied by 0.742.

The HOMA-IR was calculated using the following equation: serum glucose concentration (mM) × serum insulin concentration (µU/mL)/22.5, which was reported to have a strong correlation with the hyperinsulinemic-euglycemic clamp (*r* = 0.88). The insulin resistance for the HOMA-IR cutoff based on the ROC curve to influence the MetS risk was 2.31, but it showed low validity in the following: the AUC (0.679), sensitivity (0.645), and specificity (0.641) for the ROC using Proc logistic in SAS. The ROC showed low diagnostic ability for the MetS risk by insulin resistance [19]. Another hospital-based study in Korean adults showed that the 2.34 cutoff for HOMA-IR had 0.672 AUC, 0.628 sensitivity, and 0.657 specificities in 2006 [20]. The AUC of the ROC suggested that MetS could not estimate low- and high-insulin resistance using the HOMA-IR cutoff. MetS was defined according to the 2005 revised National Cholesterol Education Program-Adult Treatment Panel III criteria for Asia [21,22]. The results suggested that MetS did not predict the HOMA-IR risk. Therefore, a better prediction model will be needed to predict low- and high-insulin resistance using HOMA-IR. 

### 2.3. Genetic Variants for Insulin Resistance

Genotyping and quality-control processes were conducted on the DNA isolated from the peripheral blood of the participants in the Ansan/Ansung cohort by the Korean Center for Disease Control and prevention described previously in detail [18]. Genotyping was assessed using the Affymetrix Genome-Wide Human SNP array 5.0 (Affymetrix, Santa Clara, CA, USA) for the Ansan/Ansung cohort. The genetic variants were excluded when they had low genotyping accuracies (<98%), high heterozygosity (>30%), high missing genotype call rates (≥4%), or gender biases. GWAS was performed with high-insulin resistance and low-insulin resistance after adjusting for age, gender, area, and BMI using the GPLINK program version 2.0 downloaded from the website (http://pngu.mgh.harvard.edu/~purcell/plink, accessed on 14 April 2021). Fifty-two genetic variants involved with insulin resistance were selected, and ten genetic variants were selected using the genetic variant-genetic variant interaction by the GMDR program downloaded from the website (http://www.ssg.uab.edu/gmdr/, accessed on 4 May 2021). Among ten genetic variants, the best model included three genetic variants based on the trained balance accuracy (TRBA), test balance accuracy (TEBA), and cross-validation consistency (CVC) in the GMDR models [23]. The genetic variants in the best model were linked to insulin resistance, which was published in a previous study [24]. The poly-genetic risk scores (PRS) was calculated by summing the number of risk alleles in the 3-SNP model, including the slit guidance ligand 3 (*SLIT3*)_rs2974430, pleckstrin homology domain-containing A5 (*PLEKHA5*)_rs1077044, and protein phosphatase 2 regulatory subunit B-gamma (*PPP2R2C*)_rs16838853. The PRS was used to indicate the genetic impact of insulin resistance [24]. 

### 2.4. Assessment of the Food and Nutrient Intake Using Semi-Quantitative Food Frequency Questionnaires (SQFFQ) 

The usual food intake during the last six months was evaluated by SQFFQ, of which validity and reproducibility were acceptable compared with three-day records for four seasons [25]. The SQFFQ included 103 common Korean foods, and their eating frequencies were divided into the following: never or seldom, once a month, two to three times a month, one to two times a week, three to four times a week, five to six times a week, once a day, twice a day, and three times or more per day. The amount of food at each eating event was answered as more, equal, or less based on the portion size shown by the photographs of foods in each food category. The food intake of each participant was calculated by multiplying the midpoint of the selected frequencies by the selected portion size of each food. The energy and nutrients, such as protein, carbohydrates, fat and saturated, monounsaturated, and polyunsaturated fatty acids, were calculated from the food intake determined by SQFFQ, using the Can-Pro 2.0 nutrient intake assessment software developed by the Korean Nutrition Society [9]. 

### 2.5. Experimental Design for Machine Learning for Predicting Insulin Resistance by HOMA-IR

**Figure 1:**
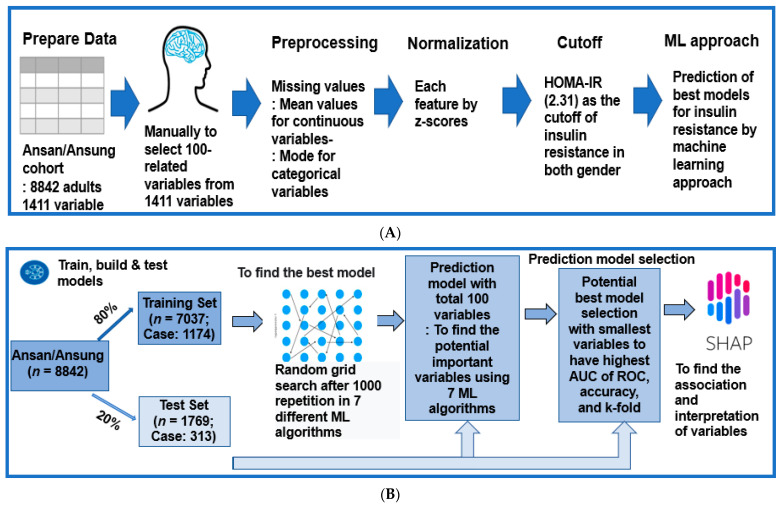
Analysis processing to generate a prediction model in the participants. (**A**) A total of 8842 adults participated, and 99 features were selected manually from 1411 in the Ansan/Ansung cohort to predict the insulin resistance model using the seven machine learning (ML) approach. Missing data were filled with the mean values for continuous variables and the mode values for the categorical variables. Data were normalized using the z-score. HOMA-IR was used as an indirect insulin resistance index, and 2.31 was used as the cutoff for participants of both genders. The prediction models for insulin resistance were generated using seven ML algorithms. (**B**) The Ansan/Ansung cohort participants were randomly divided into a training set of 80% and a test set of 20% participants. The best model was selected with a random grid search after 1000 repetitions in seven different ML algorithms, including linear regression, support vector machines (SVM), XGBoost (XGB), decision tree, random forest, K-nearest neighbor (KNN), and artificial neural network (ANN). The best prediction model was selected using the AUC of the ROC. The accuracy and k-fold cross-validation of the predicted models were assessed in the test set.

The data were curated, and 99 features potentially related to insulin resistance were selected manually from 1411 variables in the Ansan/Ansung cohort (Figure 1A). Variables with collinearity were excluded. For example, the body weight and BMI were omitted because the waist and hip circumferences, body fat percent, and muscle mass percent were included to explain the body composition. The fasting serum glucose concentrations and hemoglobin A1c (HbA1c) contents were included because they provided the different conditions of glucose homeostasis. On the other hand, the serum insulin concentrations were excluded because the HOMA-IR was an independent feature. Therefore, the HOMA-IR prediction model predicts the fasting serum insulin concentrations when the serum glucose concentrations are assigned. 

The missing values in the selected variables were filled with the mean for continuous variables and mode for categorical variables. Each variable was normalized to the z-score (Figure 1A). The training and test datasets were divided randomly into 80% (*n* = 7037) and 20% (*n* = 1769), respectively. The training set and test set included 1174 and 313 participants with high HOMA-IR (>2.31), respectively. 

In the training set, each normalized dataset of 99 features was trained to generate repeated permutations using the randomized grid search method in seven different algorithms (Figure 1B). Each algorithm found the best model to improve the area of ROC curve, accuracy, and K-fold in the test dataset. The algorithm models fitted for predicting the metabolic status were as follows: logistic regression, support vector machines (SMV), extreme gradient boosting (XGBoost), decision tree, random forest, K-nearest neighbor (KNN), and artificial neural network (ANN) [26].

### 2.6. Training for the Features for Generating Insulin Resistance Prediction Model and Testing the Models for Verifying the Prediction Model

After running the 99 features, the relative importance from the random forest and XGBoost algorithm models were used to search for the best model in the training set. The best model with the highest area of the ROC, accuracy, and K-fold in the test dataset was selected from the random forest and XGBoost algorithm models. None of the algorithm models showed a positive or negative relationship. The SHapley Additive exPlanation (SHAP; https://shap.readthedocs.io/en/latest/index.html, accessed on 16 September 2021) was used to explain the selected models from the random forest and XGBoost. 

### 2.7. Statistical Analysis

Statistical analysis was conducted using SAS (Cary, NC, USA), and a machine learning approach was performed using Scikit-learn in Python 3.8.5 (https://www.python.org/downloads/windows/, accessed on 7 October 2021) and the TensorFlow platform. The HOMA-IR cutoff was calculated using logistic regression with the ROC curve in SAS. Six prediction models for insulin resistance were generated with Scikit-learn in Python 3.8.5, while the ANN prediction model was made with the TensorFlow platform. 

The results are presented as the means ± standard deviations or number and percentage in the general characteristics of the variables. The significance of the differences between variables was determined according to genders and HOMA-IR using the two-way ANOVA in the Ansan/Ansung cohort. The statistical significance was accepted for *p*-values < 0.05. 

## 3. Results

### 3.1. Anthropometric and Biochemical Measurement of the Participants

The age of the participants was higher in women than men, but there were no significant differences in the low- (Low-IR) and high-insulin resistance (High-IR) groups. The HOMA-IR was approximately 2.6 times higher in the High-IR than Low-IR, but there was no significant difference between men and women (Table 1). Hence, a prediction model for insulin resistance according to gender is unnecessary. The anthropometric measurements, including BMI, waist circumferences, muscle mass, and fat mass, showed significant differences in gender and insulin resistance. The MetS incidence was much higher in women and high-IR groups. According to the HOMA-IR and gender, the MetS components differed significantly, but their significant differences were substantial with insulin resistance (Table 1). The serum glucose concentrations and HbA1c contents were much higher in the high-IR group than the low-IR group, while lower in women than men. The serum LDL and triglyceride concentrations showed a similar tendency to the serum glucose concentrations, while the serum HDL concentrations showed an opposite trend (Table 1). The pulse, SBP, and DBP were higher in those with insulin resistance, and the gender differences were minimal. The serum AST and ALT concentrations were also higher in the High-IR group than the Low-IR group and lower in women than men (Table 1).

### 3.2. Lifestyle-Related Variables

The energy intake based on the EER percent was similar regardless of insulin resistance, but men had a lower EER than women (Table 2). Energy and nutrient intakes showed significant differences with gender but not insulin resistance (Table 2). The CHO and fat intake were similar in the low-IR and High-IR groups, but women had a much higher carbohydrate and lower fat intake than men. The intake of saturated fatty acids (SFA), monounsaturated fatty acids (MUFA), and polyunsaturated fatty acids (PUFA) was higher in men than women, and it did not differ with insulin resistance groups (Table 2). SFA, MUFA, and PUFA intake showed similar trends with fat intake (Table 2). The differences in CHO, fat, and protein intake interacted with gender and insulin resistance: in men, their intake was higher in the high-IR group than the low-IR group, but it showed the opposite tendency in women. The protein intake also had a higher intake in men than women but was not affected with insulin resistance (Table 2). Both gender and insulin resistance status did not affect dietary fiber and calcium intake. Vitamin C and sodium intakes were affected by gender but not insulin resistance: vitamin C intake was higher, but sodium intake was lower in women than in men (Table 2).

The alcohol intake, smoking status, and regular exercise did not significantly affect insulin resistance, and only the alcohol intake was significantly different with gender (Table 2).

### 3.3. The Best Model for Explaining Insulin Resistance Using the Machine Learning (ML) Approach

**Figure 2:**
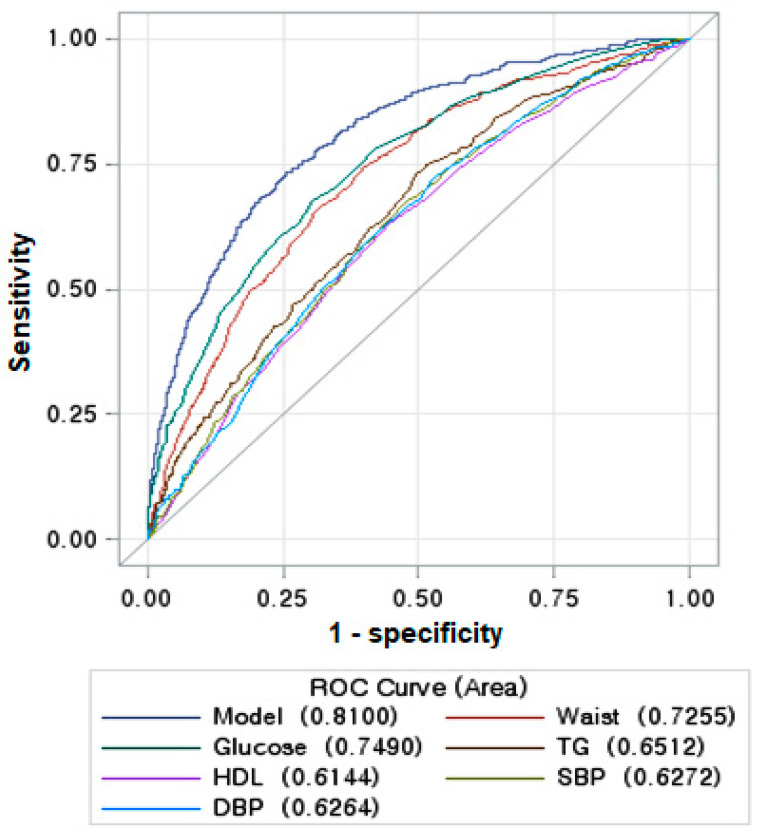
Receiver operating characteristic (ROC) curve with insulin resistance and metabolic syndrome components for the metabolic syndrome risk.

Before predicting the best model using the ML algorithm, the insulin resistance was estimated with MetS and its components. The insulin resistance was weakly linked to MetS and its components: The area of the ROC curve in the model was 0.806 (95% CI: 0.786–0.826), including waist circumferences, BMI, serum glucose, HDL, triglyceride concentrations, and blood pressure in the logistic regression model (Figure 2). The AUC of the ROC curve in each feature ranged within 0.537–0.726, and waist circumferences showed the highest AUC of the ROC curve among the features in the model. The Somer’s D (Gini) of this model was 0.613, giving it sufficient predictive power of a risk model, and the waist circumference and fasting serum glucose concentrations met the criteria (Gini > 0.4).

Ninety-nine manually selected features were applied to train the seven ML algorithms to find the optimal features for insulin resistance. The AUC of the ROC curves was 0.60–0.87, and logistic regression and XGBoost showed the highest AUC. The random forest algorithm was 0.84, and the other algorithms were less than 0.60 (Table 3). The accuracy and k-fold of all the models except the decision tree were higher than 0.8. The top 15 features were selected to predict insulin resistance from each model. The AUC with the 15 top features selected was the highest (0.85) in XGBoost, and logistic regression was higher than 0.8 in the random forest and ANN (Table 3). Furthermore, when the top important features were reduced to nine features, the AUC of the ROC with ANN increased to 0.86 from the lower AUC (0.82), while that with XGBoost and logistic regression was 0.85. The accuracy and k-fold were higher than 0.8 in all algorithms of logistic regression, XGBoost, and random forest of models with 15 and nine features (Table 3).

### 3.4. The Relative Importance of the Parameters in the Random Forest and XGBoost Prediction Models

**Figure 3:**
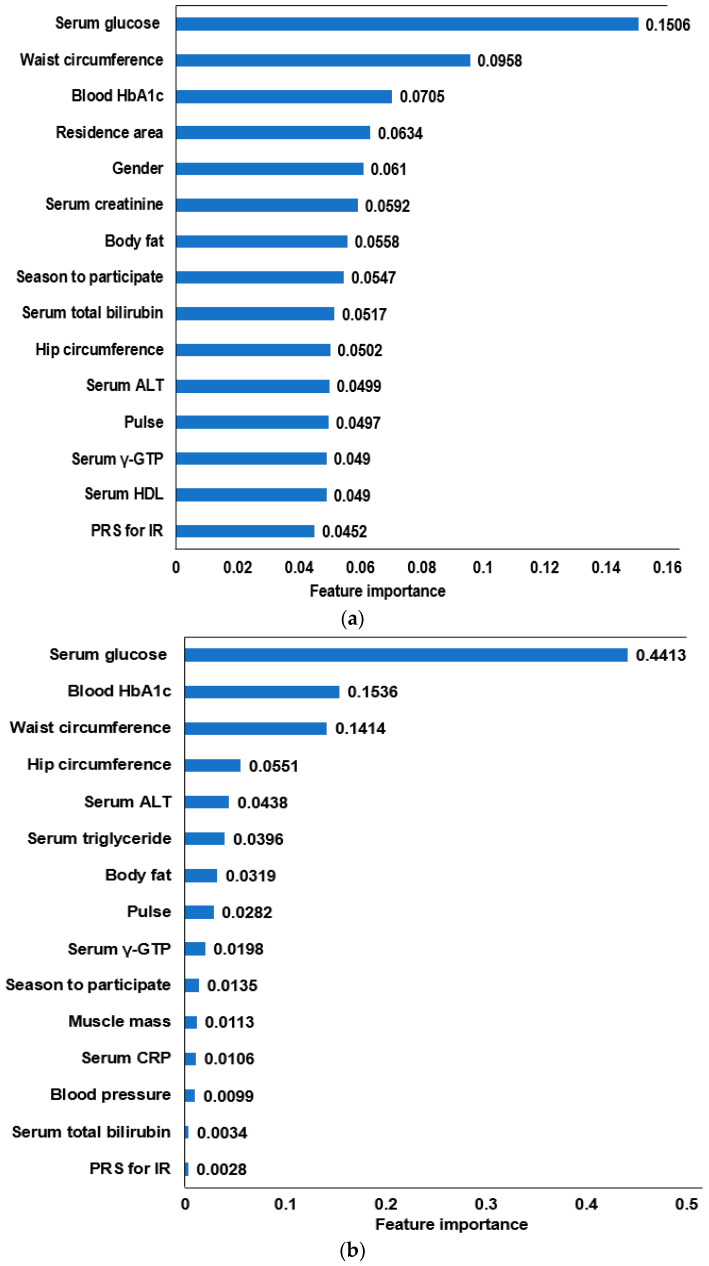
The relative importance of the top 15 features for predicting insulin resistance (IR), as determined by the XGBoost and random forest algorithms. (**a**) IR prediction model by the XGBoost algorithm 3. (**b**) IR prediction model by the random forest algorithm. ALT, alanine aminotransferase; HbA1c, hemoglobin A1c; γ-GTP, γ-glutamyl transpeptidase; HDL, high-density lipoprotein; CRP, high-sensitive C-reactive protein; PRS, polygenetic risk scores.

The AUC of the ROC curve using the XGBoost algorithm was the highest among the seven algorithms and was similar to the logistic regression algorithm. Although the AUC of the ROC was slightly lower in the random forest model than XGBoost, it met the optimal model criteria. The prediction models with relatively important features from XGBoost and random forest algorithms were obtained (Figure 3). The 15 feature models included the fasting serum glucose concentrations, waist circumferences, blood HbA1c, residence area, gender, serum creatinine, body fat, season to participate, serum total bilirubin, hip circumferences, serum ALT, pulse, serum γ-GTP, serum HDL, and genetic impact for insulin resistance in XGBoost (Figure 3a,b). In the random forest algorithms, 14 features were selected, and they were similar to the XGBoost model. On the other hand, the serum CRP concentrations, blood pressure, and muscle mass were included instead of the serum total bilirubin and creatinine concentrations. The residence area was selected from the XGBoost algorithm. Moreover, the relative importance of the features was different between the XGBoost and random forest algorithms. In the XGBoost algorithm, fasting serum glucose concentration and waist circumferences had a much larger impact on insulin resistance, but the impact of the other factors was relatively high (0.044–0.071) (Figure 3a). In the random forest algorithm, however, the fasting serum glucose concentrations, blood HbA1c, and waist circumferences mainly explained the insulin resistance; the other factors had a low impact (0.0082–0.047) on insulin resistance (Figure 3b). These differences contributed to the AUC of ROC analysis in the XGBoost and random forest algorithms. 

**Figure 4:**
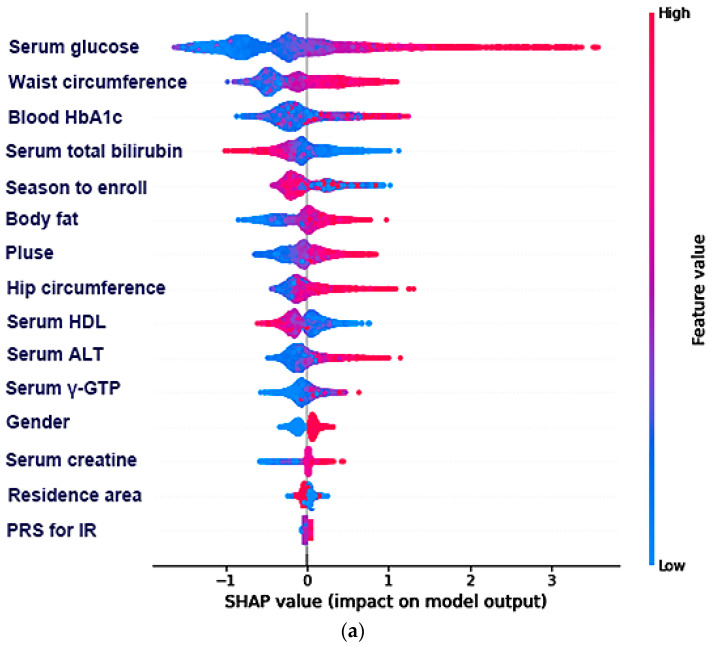

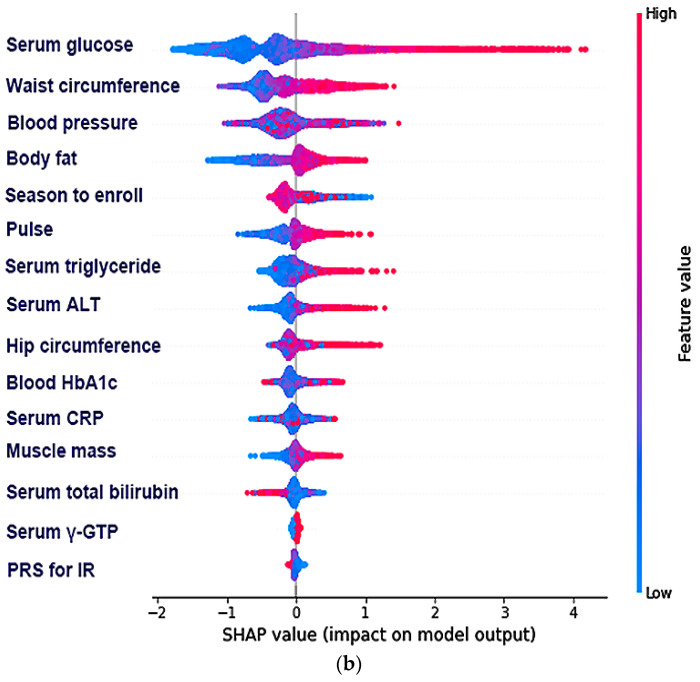
Positive and negative impact explanation of the top 15 features for predicting insulin resistance (IR) using SHAP values. (**a**) Explanation of each feature impact on the IR in the prediction model by the SHAP values in the XGBoost algorithm. (**b**) Explanation of each feature impact on the IR in the prediction model by the SHAP values in random forest algorithm. ALT, alanine aminotransferase; HbA1c, hemoglobin A1c; γ-GTP, γ-glutamyl transpeptidase; HDL, high-density lipoprotein; CRP, high-sensitive C-reactive protein; PRS, polygenetic risk scores.

The relative importance from XGBoost and the random forest did not show a positive and negative association of the selected features with insulin resistance (Figure 4). The SHAP algorithm was used to show their association using the selected features from XGBoost (Figure 4a) and random forest (Figure 4b). Most features were well separated to show the positive or negative association with insulin resistance in the SHAP values in both algorithms. However, the residential area was not separated in the positive and negative impact on insulin resistance in random forest algorithm (Figure 4a). In contrast, the serum γ-GTP concentrations and PRS for insulin resistance were not well discriminated against insulin resistance in random forest algorithm (Figure 4b).

**Figure 5:**
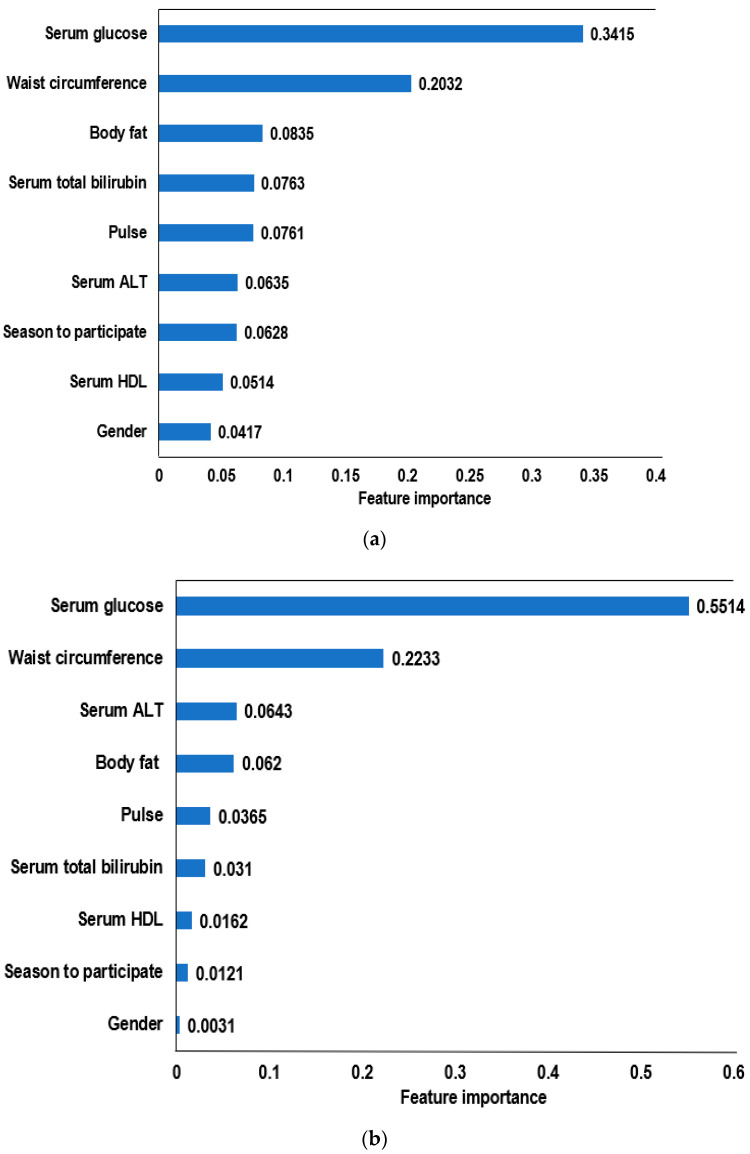

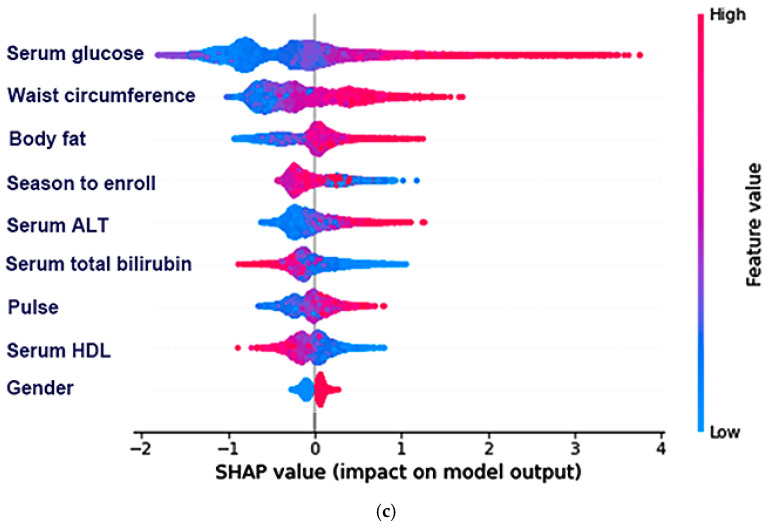
The relative importance of the top nine features for the insulin resistance (IR) prediction, as determined by the XGBoost and random forest algorithms. (**a**) IR prediction model with top 9 features using the XGBoost algorithm. (**b**) IR prediction model with top 9 features using the random forest algorithm. (**c**) Explanation of each feature impact on the IR prediction model by SHAP values using the XGBoost algorithm.

The nine feature models from XGBoost and random forest algorithms were the same. The model included the fasting serum glucose, ALT, total bilirubin, HDL concentrations, waist circumference, body fat, pulse, season to participate, and gender (Figure 5a,b). On the other hand, the relative importance of the selected features was different between the XGBoost and random forest algorithms (Figure 5a,b). SHAP shows the association of each feature to predict insulin resistance (Figure 5c).

## 4. Discussion

Insulin resistance is a common etiology of MetS. On the other hand, waist circumference (AUC of the ROC curve = 0.726) and serum glucose concentrations (AUC = 0.749) were significant components to contribute to insulin resistance among the MetS components. The other components (serum HDL and triglyceride concentrations and blood pressure) did not significantly affect insulin resistance (AUC = 0.614–0.651). Therefore, better risk factors are needed to explain insulin resistance. Furthermore, the genetic impact of insulin resistance has not been studied, and its impact on insulin resistance was investigated in the present study. 

The ML approach is an excellent way to find the risk factors and generate a prediction model. This study evaluated the best model for predicting insulin resistance using the ML approach in Korean adults aged > 40 of the Ansan/Ansung cohort. This study assessed the potential impact of the kidney and liver function in addition to obesity, glucose, and lipid metabolism that influence insulin resistance risk. Genetic and environmental factors were also considered to generate the prediction model for insulin resistance in the present study. Although PRS as the genetic impact involved in the insulin resistance risk showed a minimal impact, it was included in the 15 feature prediction model. However, environmental factors, including lifestyles, were not included in the 15 feature prediction model. It suggests that the potential genetic impact might be substantial for predicting insulin resistance risk in early life before the environmental factors involved in the prediction are developed. Therefore, people with PRS may be monitored to prevent insulin resistance in later life.

Lifestyles including nutrient intake, alcohol drinking, smoking, and physical activity, have been reported to be associated with metabolic syndrome by the imbalance between energy intake and expenditure favoring energy storage [27]. Although insulin resistance is a common underlying mechanism of metabolic syndrome, a few studies have demonstrated a direct relationship between lifestyles and insulin resistance, especially in Asians with lower insulin secretion capacity. The present showed that energy intake was higher in the high-IR group than the low-IR group in both genders, but it was not significantly different. Furthermore, nutrient intakes including carbohydrate, fat (SFA, MUFA, and PUFA), protein, vitamin C, sodium, and calcium did not differ between the low-IR and high-IR groups, but there was a gender interaction with carbohydrates, fat, and protein intake. Men tended to have a higher intake of fat and protein and a lower carbohydrate intake, but women had an opposite intake. Previous studies have demonstrated a similar result from KNHANES 2007–2009 [28]: the intakes of fat, protein, and carbohydrates (energy percent) do not differ between low-IR and high-IR groups. Energy intake was significantly lower in the high-IR group than in the low-IR group only in women [28]. In NHANES (1999–2014), vitamin C and folate intakes are inversely associated with insulin resistance [29]. Therefore, lifestyles may not be strongly and directly associated with insulin resistance to be selected as the top features for the prediction model.

ML has been used in clustering, classification, dimensionality reduction, regression, and other data mining. ML can generate a model by randomly and repeatedly learning the data in a training dataset and validating the model from a test dataset. Therefore, unlike traditional statistical programs, ML can generate a relatively accurate prediction model. The critical factors related to various diseases have been explored using ML in the medical field. In the present study, insulin resistance was divided into low- and high- groups (classification) by the cutoff (2.31) determined by the ROC curve using logistic regression. The AUC of the ROC in the prediction models might indicate good fitting. A better model than logistic regression was explored using the ML approach. The random forest and XGBoost are classification algorithms with many decision trees to generate one optimal model. Both algorithms are generally suitable for making classification models. On the other hand, they have some differences in finding the optimal models. The random forest is considered to perform bagging first. It handles overfitting, reduces variants, and uses independent classifiers [30]. By contrast, XGBoost uses the gradient boosting method to reduce bias, variance, and sequential classifiers [31]. Although XGBoost can overfit the data into the model, it reduces the disadvantages of random forest algorithms [31]. Thus, the XGBoost and random forest algorithms were used to optimize the prediction model in the present study. XGBoost exhibited a higher AUC than the random forest algorithm. The AUC of the ROC, accuracy, and k-fold in XGBoost was the highest among the seven algorithms, including the random forest algorithm used in the present study. The relative importance of the 15 features in the prediction models from XGBoost and random forest algorithms differed, and the relative importance variations in the 15 features were more considerable in the random forest algorithm than XGBoost. The top three features explained approximately 73% in the random forest model and approximately 32% in XGBoost, suggesting that approximately ten features in a random forest make a negligible contribution to the prediction model. Thus, the prediction model by the XGBoost algorithm may predict insulin resistance better.

The nine feature models from the random forest and XGBoost algorithms included the same features such as serum glucose, waist circumferences, body fat, serum ALT, serum total bilirubin, pulse, gender, and season to enroll. The Ansan/Ansung cohort participants used to predict the insulin resistance risk were middle-aged adults, and age was not included in the prediction model. Interestingly, the nine-feature model included the season to enroll in the cohort study. The SHAP algorithm explained that winter increased insulin resistance in the present study. Hence, winter had a higher insulin resistance risk than summer. Previous studies reported that the seasonality of insulin resistance is linked to age and caused by impaired thermoregulation [32]. In the Rotterdam Study, the middle-aged and elderly had seasonal variations for insulin resistance determined by the HOMA-IR, and the elderly persons have higher seasonal variation for insulin resistance (0.29 units: 95% CI: 0.21, 0.37) than the middle-aged adults (0.11 units; 95% CI: 0.03, 0.20) [32,33]. In older men, the seasonal variations for insulin resistance were also shown with a hyperinsulinemic-euglycemic clamp [34]. Consistent with the present study, previous studies reported the winter peak of insulin resistance in middle-aged and older adults [32,33].

As expected, the prediction model of insulin resistance included waist circumferences, body fat, and serum HDL concentrations, but it did not contain the serum triglyceride and blood pressure. Although blood pressure was not included in the prediction model, the pulse might explain blood pressure status. The heart rate is positively associated with the risk of blood pressure, hypertension, and cardiovascular disease [35]. Nevertheless, the resting heart rate is linked to increased arterial stiffness, particularly in persons with increased aortic stiffness, regardless of the blood pressure [36]. Furthermore, insulin resistance, in addition to obesity, influences heart rate [37]. The prediction model with 15 features contained the blood pressure and pulse features. The pulse showed higher relative importance scores in the random forest algorithm but not in the XGBoost algorithm. Thus, the pulse can be an indicator of blood pressure. Because people can easily count their pulse and measure waist circumference, they can be used to check insulin resistance in daily life.

The nine feature-prediction models for insulin resistance risk included the serum ALT and total bilirubin concentrations, indicating that insulin resistance was closely linked to a liver function to induce NAFLD and NASH [38]. Reducing insulin resistance has shown some alleviation of NAFLD [38]. The liver function is not included in the MetS definition, but a liver dysfunction has emerging evidence associated with insulin resistance risk, although it debates whether insulin resistance is a cause or effect of NAFLD [39]. Insulin resistance is interrelated to develop and exacerbate NAFLD and NASH, and reducing insulin resistance can alleviate the diseases [40]. In prospective studies, elevated serum ALT and γ-GTP concentrations were independent predictors of MetS, type 2 diabetes, and cardiovascular diseases [41]. Furthermore, the linking mechanism between insulin resistance and liver disorder is related to the modulated rates of adipose tissue lipolysis and de novo lipogenesis, changed fat distribution, impaired mitochondrial fatty acid β-oxidation, modulated adipokines, and cytokines concentration. Thus, the liver dysfunction represents the elevated liver enzymes, ALT, ALT/AST, and γ-GTP in the circulation [39]. However, liver enzyme concentrations alone should not be used as a surrogate marker for NAFLD and NASH since some patients with these diseases have normal AST and ALT concentrations in the circulation [42]. Therefore, the serum ALT concentration can be added to predict the insulin resistance risk, but adults with normal serum ALT concentration also need to be watched if they have other risk factors. The serum total bilirubin concentrations in the present study were inversely associated with the insulin resistance risk. Previous studies also reported that serum total bilirubin concentrations are inversely related to the MetS risk in various ethnic groups [43,44,45,46]. It might be associated with the cholesterol metabolism in the liver. 

The strength of the study was novel to show that the poly-genetic variants belonged to the 15-feature prediction model when the environmental factors, including nutrient intake and lifestyles, were not included. Pulse and seasons with other medical health-checkup were included in the 9-feature model, and they can be easily implicated into the smartwatch to check insulin resistance and provide a health-related personal warning daily. This study had some limitations. The data originated from a cross-sectional study, and hence, the results cannot be explained as a cause-and-effect association. The study population was Koreans aged ≥ 40 years, which cannot be extended to adolescents and young adults. This study has the strength to generate an optimal prediction model to explain insulin resistance by metabolic features and genetic factors. The metabolic features included the previously designated ones with new ones added. The liver function index such as serum ALT and total bilirubin concentrations should be considered to predict insulin resistance. 

## 5. Conclusions

**Figure 6:**
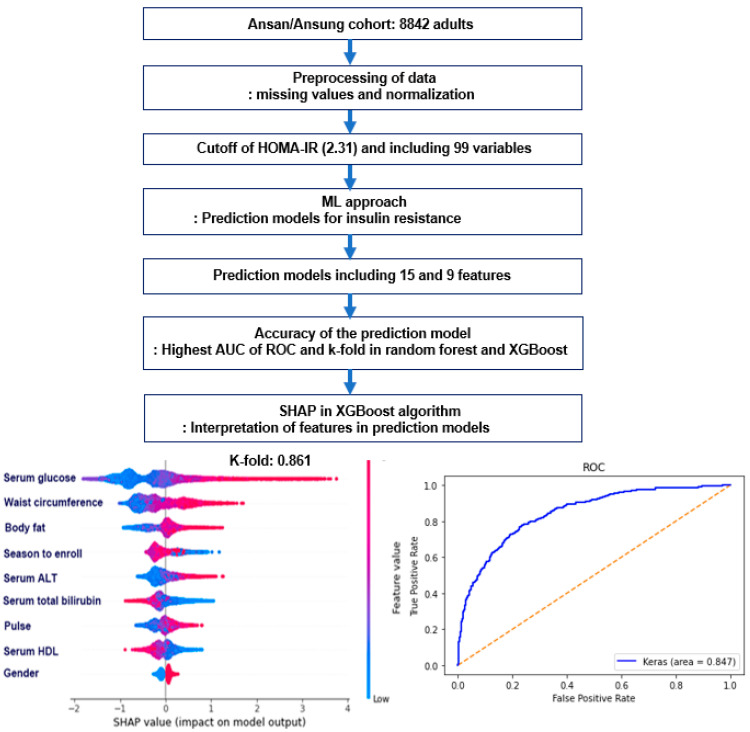
Summary of the main findings to explore the prediction model for insulin resistance. HOMA-IR, homeostasis model assessment of insulin resistance.

XGBoost, logistic regression, random forest, and ANN algorithms generated the optimal prediction model for insulin resistance among seven ML-based approaches, summarized in Figure 6. The prediction model with 15 features included metabolic and genetic factors but not food intake and lifestyles in the XGBoost and random forest algorithms. Although they included different features, the prediction model with XGBoost and random forest showed good validation. However, the nine feature prediction models included the same, but their relative importance differed. The models included the fasting serum glucose, ALT, total bilirubin, HDL concentrations, waist circumference, body fat, pulse, season to participate, and gender. In conclusion, liver function, pulse, and seasonal variation in addition to MetS components should be considered to predict insulin resistance in Koreans aged over 40. The ML algorithms, particularly XGBoost, logistic regression, random forest, and ANN, can help find risk factors for various diseases and predict the disease in a clinical setting.

## Figures and Tables

**Table 1:** Characteristics of the participants in the Ansan/Ansung cohort.

	Men (*n* = 4183)		Women (*n* = 4659)	
	Low-IR (*n* = 3906)	High-IR (*n* = 677)	Low-IR (*n* = 3850)	High-IR (*n* = 809)
Age (year)	52.0 ± 0.15 ^b^	50.6 ± 0.34 ^c^	52.4 ± 0.14 ^a^	53.7 ± 0.31 ^a^***
HOMA-IR	1.22 ± 0.03 ^c^	3.43 ± 0.08 ^a^	1.37 ± 0.03 ^b^	3.41 ± 0.07 ^a^**^###^
BMI (mg/kg^2^)	24.0 ± 0.06 ^d^	26.2 ± 0.13 ^b^	24.5 ± 0.05 ^c^	26.7 ± 0.12 ^a^***^###^
Waist circumferences(cm)	82.2 ± 0.21 ^c^	88.4 ± 0.54 ^a^	80.3 ± 0.22 ^d^	86.5 ± 0.46 ^b^***^###^
Skeletal muscle mass index (%)	35.4 ± 0.04 ^a^	33.9 ± 0.10 ^b^	30.8 ± 0.04 ^c^	29.4 ± 0.09 ^d^***^###^
Fat mass (%)	21.3 ± 0.09 ^d^	24.8 ± 0.21 ^c^	31.3 ± 0.09 ^b^	34.4 ± 0.20 ^a^***^###^
MetS (%)^9^	558 (15.9)	256 (37.8) ***	813 (21.1)	350 (43.3) ***
Serum glucose (mg/dL)	86.0 ± 0.34 ^c^	112.4 ± 0.77 ^a^	81.7 ± 0.33 ^d^	101.2 ± 0.70 ^b^***^###^
HbA1c (%)	5.71 ± 0.15 ^c^	6.44 ± 0.04 ^a^	5.69 ± 0.15 ^c^	6.30 ± 0.03 ^b^**^###^
Serum total cholesterol (mg/dL)	190 ± 0.61 ^b^	199 ± 1.38 ^a^	190 ± 0.58 ^b^	199 ± 1.26 ^a###^
Serum HDL (mg/dL)	44.1 ± 0.17 ^b^	41.0 ± 0.39 ^d^	46.1 ± 0.16 ^a^	43.0 ± 0.35 ^c^***^###^
Serum LDL (mg/dL)	105 ± 0.83 ^c^	103 ± 2.2 ^c^	113 ± 0.85 ^b^	118 ± 1.83 ^a^***
Serum Triglyceride (mg/dL)	169 ± 1.74 ^c^	227 ± 3.96 ^a^	142 ± 1.66 ^d^	183 ± 3.62 ^b^***^###^
Serum CRP (mg/dL)	0.24 ± 0.01	0.29 ± 0.02	0.21 ± 0.01	0.26 ± 0.02
Pulse	62.8 ± 0.13	64.8 ± 0.29	64.0 ± 0.12	67.3 ± 0.27 ***^###^
SBP (mmHg)	119 ± 0.46 ^b^	125 ± 1.18 ^a^	119 ± 0.47 ^b^	127 ± 1.01 ^a###^
DBP (mmHg)	76.5 ± 0.27	80.7 ± 0.70	74.9 ± 0.28	80.0 ± 0.60 **^###^
Serum AST (U/L)	32.4 ± 0.31 ^b^	34.5 ± 0.70 ^a^	27.0 ± 0.29 ^c^	28.0 ± 0.64 ^c^***^##^
Serum ALT(U/L)	31.8 ± 0.45 ^b^	43.6 ± 1.02 ^a^	22.4 ± 0.43 ^d^	27.8 ± 0.94 ^c^***^###^

Low-IR, low insulin resistance (≤2.31 HOMA-IR); High-IR, high insulin resistance (>2.31 HOMA-IR). HOMA-IR, homeostasis model assessment of insulin resistance; BMI, body mass index; HbA1c, hemoglobin A1c; HDL, high-density lipoprotein; LDL, low-density lipoprotein CRP, high-sensitive C-reactive protein; SBP, systolic blood pressure; DBP, diastolic blood pressure; AST, aspartate aminotransferase; ALT, alanine aminotransferase. Skeletal muscle mass index was calculated by dividing skeletal muscle mass by body weight × 100. * significantly different by genders at *p* < 0.05, ** at *p* < 0.01, *** at *p* < 0.001. ## significantly different by HOM-IR at *p* < 0.01, ### at *p* < 0.001. ^a,b,c^ Different superscript letters of the means indicate significant differences among the groups by Tukey’s test at *p* < 0.05.

**Table 2:** Nutrient intake and lifestyle-related variables.

	Men (*n* = 4183)		Women (*n* = 4659)	
	Low-IR (*n* = 3906)	High-IR (*n* = 677)	Low-IR (*n* = 3850)	High-IR (*n* = 809)
Energy (EER%)	96.8 ± 0.66 ^b^	97.1 ± 1.49 ^b^	106 ± 0.62 ^a^	109 ± 1.38 ^a^***
CHO (En%)	69.7 ± 0.12 ^b^	68.8 ± 0.27 ^b^	71.7 ± 0.11 ^a^	72.4 ± 0.25 ^a^***^++^
Fat (En%)	15.3 ± 0.09 ^a^	15.9 ± 0.21 ^a^	13.6 ± 0.09 ^b^	13.0 ± 0.19 ^c^***^++^
SFA (En%)	3.76 ± 0.04 ^a^	3.96 ± 0.09 ^a^	3.15 ± 0.04 ^b^	2.93 ± 0.08 ^b^***^++^
MUFA (En%)	4.88 ± 0.04 ^a^	5.04 ± 0.09 ^a^	4.00 ± 0.04 ^b^	3.76 ± 0.09 ^b^***^++^
PUFA (En%)	2.29 ± 0.02 ^a^	2.37 ± 0.04 ^a^	1.94 ± 0.02 ^b^	1.90 ± 0.03 ^b^***^+^
Protein (En%)	13.7 ± 0.04 ^b^	14.1 ± 0.09 ^a^	13.5 ± 0.04 ^c^	13.4 ± 0.09 ^c^***^++^
Dietary fiber (g)	6.92 ± 0.08	7.11 ± 0.16	7.16 ± 0.07	7.31 ± 0.15
Vitamin C (mg)	121 ± 2.17 ^b^	126 ± 4.57 ^b^	136 ± 2.09 ^a^	141 ± 4.30 ^a^***
Calcium (mg)	486 ± 5.86	481 ± 12.3	482 ± 5.63	477 ± 11.6
Sodium (g)	3.37 ± 0.04 ^a^	3.39 ± 0.07 ^a^	3.02 ± 0.03 ^b^	3.03 ± 0.07 ^b^***
Alcohol intake (g/day)	19.1 ± 0.35^a^	19.5 ± 0.80 ^a^	1.29 ± 0.33 ^b^	1.48 ± 0.73 ^b^***
Smoking				
Former smoker	166 (4.8)	33 (4.9)	46 (1.22)	13 (1.65)
Smoker	1567 (44.9)	298 (44.1)	86 (2.28)	19 (2.41)
Regular exercise (yes, %)	1043 (73.9)	144 (68.3)	933 (71.9)	213 (77.2)

EER, energy estimated requirement; CHO, carbohydrate; En%, energy percent. SFA, saturated fatty acids; MUFA, monounsaturated fatty acids; PUFA, polyunsaturated fatty acids. *** Significantly different by genders at *p* < 0.001. + significant interaction between gender and HOMA-IR at *p* < 0.05 and ++ at *p* < 0.01. ^a,b,c^ Different superscript letters of the means indicate significant differences among the groups by Tukey’s test at *p* < 0.05.

**Table 3:** The area under the curve (AUC) of the receiver operating characteristic (ROC) curve, accuracy, and k-fold of prediction models generated from machine-learning algorithms in the Ansan/Ansung cohort.

99 Features	Logistic Regression	XGBoost	Decision Tree	KNN	SVM	Random Forest	ANN
AUC of ROC	0.866 (0.865–0.867)	0.866 (0.865–0.867)	0.647 (0.646–0.647)	0.662 (0.661–0.663)	0.597 (0.596–0.597)	0.836 (0.835–0.836)	0.816
Accuracy	0.867 (0.867–0.868)	0.868 (0.868–0.869)	0.793 (0.792–0.793)	0.826 (0.825–0.827)	0.859 (0.858–0.859)	0.841 (0.840–0.841)	
k-fold	0.858 (0.853–0.863)	0.859 (0.856–0.863)	0.786 (0.764–0.786)	0.821 (0.818–0.825)	0.851 (0.848–0.854)	0.833 (0.831–0.834)	
Top 15 features							
AUC of ROC	0.849 (0.848–0.850)	0.853 (0.853–0.854)	0.639 (0.638–0.640)	0.694 (0.693–0.695)	0.574 (0.574–0.575)	0.831 (0.830–0.832)	0.822
Accuracy	0.868 (0.867–0.868)	0.877 (0.876–0.877)	0.798 (0.797–0.798)	0.837 (0.836–0.837)	0.855 (0.854–0.856)	0.860 (0.859–0.860)	
k-fold	0.856 (0.850–0.862)	0.861 (0.853–0.870)	0.777 (0.768–0.785)	0.827 (0.818–0.831)	0.850 (0.846–0.852)	0.856 (0.853–0.859)	
Top 9 features							
AUC of ROC	0.849 (0.848–0.850)	0.853 (0.852–0.853)	0.636 (0.635–0.636)	0.691 (0.690–0.692)	0.561 (0.560–0.561)	0.836 (0.835–0.837)	0.862
Accuracy	0.867 (0.867–0.868)	0.868 (0.867–0.868)	0.791 (0.790–0.792)	0.834 (0.833–0.834)	0.853 (0.852–0.853)	0.862 (0.862–0.863)	
k-fold	0.856 (0.851–0.861)	0.861 (0.857–0.864)	0.779 (0.764–0.795)	0.828 (0.824–0.835)	0.848 (0.843–0.853)	0.857 (0.853–0.859)	

Prediction models were generated from the training set with 80% of the Ansan/Ansung cohort, and its 20% was used as a test set. KNN, K-Nearest Neighbor; SVM, support vector machine; ANN, artificial neural network. The top 15-feature prediction model generated from XGBoost included serum glucose, waist circumference, blood HbA1c, serum total bilirubin, season to enroll the study, body fat, pulse, hip circumference, serum HDL, ALT, and γ-GTP, gender, serum creatinine, residence area, and PRS for insulin resistance. The top 9-feature prediction model generated from XGBoost contained serum glucose, waist circumference, body fat, serum ALT, serum total bilirubin, pulse, serum HDL, and gender.

## Data Availability

The data presented in this study are available on request from the corresponding author.
